# The role of noise in self-organized decision making by the true slime mold *Physarum polycephalum*

**DOI:** 10.1371/journal.pone.0172933

**Published:** 2017-03-29

**Authors:** Bernd Meyer, Cedrick Ansorge, Toshiyuki Nakagaki

**Affiliations:** 1 Faculty of Information Technology, Monash University, Melbourne, Victoria, Australia; 2 Institute for Geophysics and Meteorology, University of Cologne, Cologne, Germany; 3 Research Institute for Electronic Science, Hokkaido University, Sapporo, Japan; Georgia State University, UNITED STATES

## Abstract

Self-organized mechanisms are frequently encountered in nature and known to achieve flexible, adaptive control and decision-making. Noise plays a crucial role in such systems: It can enable a self-organized system to reliably adapt to short-term changes in the environment while maintaining a generally stable behavior. This is fundamental in biological systems because they must strike a delicate balance between stable and flexible behavior. In the present paper we analyse the role of noise in the decision-making of the true slime mold *Physarum polycephalum*, an important model species for the investigation of computational abilities in simple organisms. We propose a simple biological experiment to investigate the reaction of *P. polycephalum* to time-variant risk factors and present a stochastic extension of an established mathematical model for *P. polycephalum* to analyze this experiment. It predicts that—due to the mechanism of stochastic resonance—noise can enable *P. polycephalum* to correctly assess time-variant risk factors, while the corresponding noise-free system fails to do so. Beyond the study of *P. polycephalum* we demonstrate that the influence of noise on self-organized decision-making is not tied to a specific organism. Rather it is a general property of the underlying process dynamics, which appears to be universal across a wide range of systems. Our study thus provides further evidence that stochastic resonance is a fundamental component of the decision-making in self-organized macroscopic and microscopic groups and organisms.

## 1 Introduction

Self-organization enables even simple organisms to solve surprisingly complex tasks, specifically optimization tasks essential for survival [1]. Prominent examples are ant colonies which optimize their foraging choices among multiple food patches [2] taking a variety of criteria into account [3] and slime molds, which optimize path choices even in complex mazes [4].

In the past, the self-organized behavior of such organisms has mostly been investigated in unchanging, static environments. While this seems a natural starting point for such investigations, dynamic settings are much more relevant to the behavior of organisms in the real world, where change is ubiquitous. This is why recently the focus of research has been shifting towards dynamic environments where the properties of the environment change over time. The question addressed is “can species *x* efficiently adapt its behavioral patterns to the environmental changes?”

Such a dynamic setting imposes additional burdens on a systematic investigation. First, the notion of optimality becomes even more slippery than it is in the static case already [5]. Second, the corresponding experimental set-ups are more complex, leading to an increase in the number of parameters governing numerical studies. Thus, theoretical research guiding the design of meaningful (and realistically feasible) experiments becomes very important. Here we present a theoretical study that analyzes fundamental properties of dynamic decision making by the true slime mold *Physarum polycephalum*, an important model species for the study of information processing in biological systems [6, 7]. Our study directly suggests foraging experiments that will allow us to verify these properties for the real system.

### 1.1 Noise-induced adaptive decision-making

One of the recent advances in research into dynamic foraging was the finding that noise in the decision making process is one of the crucial factors enabling self-organized insect societies to adapt their foraging patterns to changes in the environment [8, 9]. This interesting and counter-intuitive result suggests that noise is not a disturbance in self-organized systems. On the contrary, noise serves an important functional role. The studies [8, 9] are based on experiments with mass foraging ant colonies, one of the prototypical model systems in the study of self-organization. Interestingly, the fact that noise enables adaptive decision making is not due to any specific physical details of the ant foraging mechanism. Instead, it arises from very general mathematical properties of the underlying self-organized processes [10]. This suggests that the same should also apply to other similar types of self-organized collective decision making in organisms such as slime molds and bees.

In the present paper we investigate the assessment of time-variant risk for the true slime mold *P. polycephalum*. We show that noise can enable *P. polycephalum* to correctly assess time-variant risk factors in dynamic environments and, as a consequence, to make near-optimal foraging choices. We extend a deterministic phenomenological model developed by Tero *et al.* [11] for the foraging behavior of *P. polycephalum* to explicitly capture effects of noise. Numerical and analytical investigation of the resulting stochastic model shows that a well-attuned level of noise can enable *P. polycephalum* to integrate variable risk factors correctly over time. This is not the case if there is little or no noise in the system. We suggest comparatively simple biological experiments that will allow us to test these predictions.

Our results hold interest beyond their immediate relevance for the study of *P. polycephalum*. They demonstrate with a concrete case that it is possible to transfer insights about collective behavior from one species (ants) to another species (slime molds). Ants and slime molds are physically entirely different systems. Yet, the self-organized mechanisms that govern their fundamental behavior are so similar that both species share essential behavioral characteristics.

Most importantly, the fundamental mathematical structure of the self-organized decision making mechanisms in these systems is similar to those in a broad variety of other organisms, such as bees [1] and bacteria [12], to those in human social decision making [13, 14], and even to those in bio-inspired engineering solutions, such as swarm robots [15]. In conjunction with earlier work on similar phenomena in ant colonies our study thus provides further evidence that noise may play a crucial role in the decision making of a broad range of self-organized systems beyond those investigated so far.

### 1.2 Path finding by *P. polycephalum*

*P. polycephalum* is a slime mold that spends most of its life cycle as a plasmodium, a uni-cellular multinucleate amoeboid. The plasmodium is an aggregate of protoplasm with a network of tubular elements. The protoplasm is differentiated into two phases: a gel phase (ectoplasm) that makes up the walls of the tubular structures, and a sol phase (endoplasm) that flows within the tubes. The motion of the sol, so-called shuttle streaming, is driven by organized rhythmic contractions of the gel with a period of ca. two minutes. The sol serves as a circulation system for the cell transporting nutrients and chemical signals. The tubes act as pseudopodia and enable the organism to navigate around its environment [4, 16]. The organism can reconfigure the tube network within a few hours in response to changes in external conditions. As it moves over a surface, the plasmodium changes its shape and if food is placed at different points, it will put out tubes that connect these food sources [6].

It has been shown that *P. polycephalum*, despite its extremely simple morphology, is able to solve computational problems of surprising complexity. About a decade ago, a seminal experiment [4] demonstrated that *P. polycephalum* can solve the shortest path problem in mazes. Since then, these studies have been extended and it has been demonstrated that it can solve (or approximately solve) a variety of other network optimization problems [7, 17, 18] even when taking multiple objectives into account [19]. It has also been shown that *P. polycephalum* possesses a memory and is able to anticipate periodic events [20]. These capabilities in combination with its simple morphology and comparatively large size make *P. polycephalum* an almost ideal model system for the study of information processing and problem solving in biological systems.

**Fig 1:**
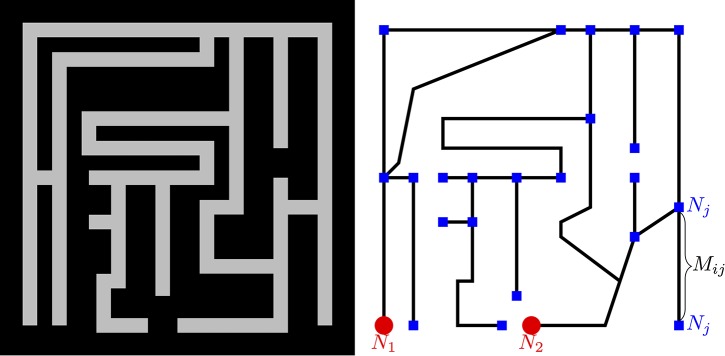
Maze solving by *P. polycephalum* following [4]. Panel (a) shows a schematic of the set-up used by [4], where black color corresponds to inhabitable space for *P. polycephalum*. Panel (b) shows the abstract graph model of the maze in (a) with nodes in black and edges in blue color. The two food sources *N*_1_, *N*_2_ are shown in red.

The most fundamental demonstration of its computational abilities is the maze experiment [4]. In this experiment an agar surface is masked with plastic film, such that the accessible surface forms a maze. Food (oat flakes) is placed at the entrance and exit points of the maze, and pieces of the plasmodium are distributed in the maze. These pieces spread and coalesce to form a single plasmodium that fills the agar maze and avoids the dry surface of the plastic film. Subsequently the plasmodium shrinks and only leaves a single thick tube behind which traces the shortest path between the two food sources (see Fig 1).

It appears this is the result of the organism’s attempt to simultaneously optimize two different goals, namely to (1) maintain sufficient connectivity of the entire plasmodium in order to maintain chemical communication, and (2) to maximize food absorption [19]. The maximization of both goals causes almost all body mass to cover the two food sources while only a minimal connection between these areas is maintained.

While a full explanation of these abilities from first bio-physical principles is currently still beyond reach, Tero *et al.* have proposed a simple phenomenological model that describes the development of the tube network [11]. We use this model as the departure point for our analysis of risk evaluation by *P. polycephalum*.

## 2 Methods

### 2.1 The deterministic Tero-Kobayashi model

In the Tero–Kobayashi model, the shape of the cell body is represented by a graph: the edges correspond to tubes and nodes correspond to the junctions between tubes. Fig 1(d) shows the example graph for the maze-solving experiment. The two nodes with food-sources are labeled *N*_1_ and *N*_2_ and the other nodes are numbered *N*_3_, *N*_4_, *N*_5_, …. Edges (tubes) between node *i* and *j* are labeled *M*_*i*,*j*_. Suppose that the fluid (sol) pressure at nodes *i* and *j* is *p*_*i*_ and *p*_*j*_, respectively, and that the tubes are idealized as cylinders of length *L*_*i*,*j*_ and radius *r*_*i*,*j*_. Assuming a Poiseuille flow, the flux through the tube is

$$\begin{array}{c} Q_{i,j}=\frac{8\pi r^{4}\left(p_{i}-p_{j}\right)}{\eta L_{i,j}}=\frac{D_{i,j}}{L_{i,j}}\left(p_{i}-p_{j}\right) \end{array}$$ (1)

where *η* is the viscosity of the fluid (sol), and $D_{i,j}=\frac{8\pi r^{4}}{\eta }$ is a measure of the conductivity of the tube. Although the tube walls are not rigid and the radius changes over time, the dynamics of tube adaptation are slow enough (10-20 minutes) for the flow to be taken as steady in time. The amount of fluid at internal nodes must be conserved, while the nodes that correspond to food sources drive the flow through the network by changing their volume, so that

$$\begin{array}{c} \sum_{j}Q_{i,j}=\{\begin{array}{ccc} 0 & \text{if} & i\neq 1,2\\ S_{i} & \text{otherwise} \end{array} \end{array}$$ (2)

with *S*_1_ + *S*_2_ = 0, because the total volume of fluid in the network is conserved. The source terms *S*_*i*_ could be periodic in time and drive shuttle streaming through the network. However, because the time scale of network adaptation is an order of magnitude longer than the time scale of shuttle streaming, the sources are taken to be constant in the following.

In *P. polycephalum* the radii of the tubes change in response to the sol flux: while generally all tubes have a tendency to shrink, tubes with a large flow expand in response to the flow. Thus the evolution of tube conductivities can be modelled as:

$$\begin{array}{c} \frac{\partial D_{i,j}}{\partial t}=f(|Q_{i,j}\left|\right)-\delta D_{i,j} \end{array}$$ (3)

With initial conditions *D*_*i*,*j*_(*t* = 0) to be specified. This establishes a self-limiting feedback system in which positive feedback is counterbalanced by negative feedback (shorter and larger tubes attract more flow, which in turn expands the tubes, while longer and less used tubes shrink and thus attract even less flow). The evolution of the tubular network according to this model agrees well with biological experiments [11]. A biologically plausible choice for *f* is a sigmoidal function. To make our results comparable with earlier literature we choose the following form which has been used to analyse the Tero-Kobayashi model in previous work [11]:

$$\begin{array}{c} f_{\left[\alpha ,\gamma ,\mu \right]}\left(Q\right)=\left(\gamma +\alpha \right)\frac{Q^{\mu }}{\gamma +\alpha Q^{\mu }} \end{array}$$ (4)

While the forcing function *f* appears as a three-parametric function (parameters are *α*, *γ* and *μ*) of the dependent variable *Q*, it is only the linear combination *γ*/*α* which has an impact on the forcing. This symmetry is exploited by rewriting *f* as a two-parametric function reducing the number of parameters on which the system depends, by one:

$$\begin{array}{c} f_{\left[\epsilon ,\mu \right]}\left(Q\right)=\left(1+\epsilon \right)\frac{Q^{\mu }}{\epsilon +Q^{\mu }}\text{;}\;\text{with}\;\epsilon \equiv \gamma /\alpha . \end{array}$$ (5)

Hence, a specific value of *ϵ* incorporates all combinations of *α* and *γ* which yield this specific value.

Note that this is a saturating feedback function. While it complicates analytical investigation of the system, a saturating function is biologically more realistic. In the following we use Eq (4) with *μ* = 2 and *ϵ* = 0.2 unless stated otherwise. The choice of *μ* = 2 is motivated by comparability with previous work. The parameter *ε* is chosen such that the system is well within its tristable regime. Backed by the extensive analytical investigation of the system in Appendix A, we are confident that the dynamics of the system depend on the value of *ε* only in quantitative details, but not qualitatively.

**Fig 2:**
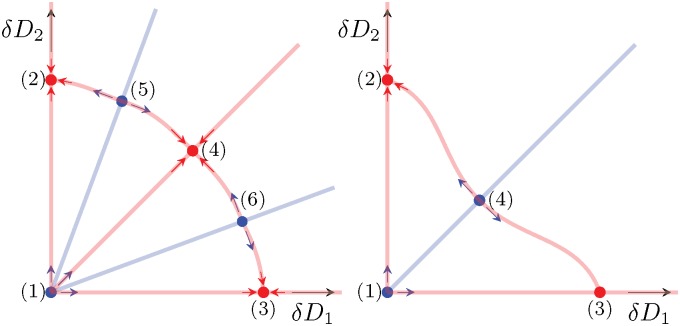
Schematic of the *δD*_1_–*δD*_2_ parameter space. (a) for *ϵ* < 1/4 and (b) for *ϵ* > 1/4. Stable equilibria are marked by a red circle, unstable ones by a blue circle; linear stability of the respective equilibria is also indicated by arrows surrounding them. The equilibria are numbered as in Appendix A. Trajectories in the *D*_1_–*D*_2_ phase-space cannot cross any of the lines drawn; hence the lines divide the phase space into 8 sectors in which trajectories stay for all times. The blue lines are given by *D*_2_ = *a*_2,3_*D*_1_ and divide the basins of attraction of the three (*ϵ* < 1/4) respectively two (*ϵ* > 1/4) fix points.

To gain an understanding of the system dynamics, we consider the simplest possible decision network consisting of only two different paths between two food sources. Dropping subscripts *i*, *j* for nodes and simply numbering the two tubes as *i* ∈ {1, 2}, the system becomes

$$\begin{array}{c} \frac{\partial D_{i}}{\partial t}=-\delta D_{i}+f_{\epsilon }(\frac{D_{i}/L_{i}}{D_{1}/L_{1}+D_{2}/L_{2}}) \end{array}$$ (6)

with initial conditions *D*_1_(*t* = 0) ≡ *D*_1,0_ and *D*_2_(*t* = 0) ≡ *D*_2,0_. In Appendix A we show that dynamics of this system critically depend on *ϵ*. For *ϵ* < 1/4 it can be shown that the system has two unstable and three stable equilibria (Fig 2). Two of the stable equilibria correspond to full convergence to a single tube (i.e. a single foraging path) (*D*_1_ = 0 or *D*_2_ = 0) and the third one corresponds to equal utilization of both paths *D*_1_ = *D*_2_ ≠ 0. As *ϵ* → 1/4 the basin of attraction for the third fixpoint vanishes and the fixpoint becomes unstable for *ϵ* < 1/4. Thus, depending on the parameter *ϵ*, the system is either a binary or a ternary decision model.

### 2.2 Dynamic path finding: A thought experiment

Our fundamental concern is whether self-organized decision making enables *P. polycephalum* to successfully react to dynamically changing environments. The maze experiment reviewed above investigates a static scenario. The question arises whether it can be adequately modified into a dynamic version. A commonly used set-up for dynamic foraging experiments is to change the type or location of food sources or the paths to these. However, as the body of *P. polycephalum* actually covers the food sources and the paths, this is difficult to achieve without disturbing the organism too much. The possibility of an alternative set-up arises as *P. polycephalum* exhibits phototaxis, being photophobic at some wavelengths in the visible range of EM radiation [21]. The organism experiences bright light as a “risk” factor and consequentially tends to withdraw its tubes in more brightly lit areas. In the model this can be captured by increasing the thinning factor *δ*. It has been shown experimentally that *P. polycephalum* is able to select paths in inhomogeneously lit fields such that the risk imposed by light and path lengths, respectively, are balanced [19]. This photophobic behavior of the organism suggests a different set-up for a dynamic foraging experiment: instead of changing the spatial arrangement we can change the lighting on different parts of the set-up, thus avoiding to disturb the organism in the process. Toxic light has also been used to induce a time variant risk in [22]. However, the focus of [22] was on spatial search rather than on the role of noise in a fundamental binary decision task, which we investigate here.

We propose the following dynamic foraging experiment to clarify how the self-organized organism can successfully cope with changing environments. *P. polycephalum* is presented with a minimal “maze” of only two alternative paths connecting two food sources. Both paths have the same length and are illuminated with light sources of the same calibrated luminosity. The reduction to only two possible paths is made to ensure that the mathematical model remains tractable. So far there is no reason for the organism to prefer either path. From experiments and the analysis of the basic model [11] we know that the organism will select one of the two paths depending on which one is initially set up with the thicker tube (note that we can regulate the initial tube thickness according to requirements by preceding the experiment with an initialization phase in which targeted lighting is used to thin selected tubes). Now consider the use of intermittent lighting instead of continuous lighting. Conceptually, if both paths are lit with the same luminosity, but with different light-dark periods, one of them should be selected preferentially, because it represent the lower total risk (integrated over time). Number the two paths 1 and 2 and let the light-to-dark ratio on these be $r_{i}=\frac{l_{i}}{d_{i}}$, where *l*_*i*_ and *d*_*i*_ are the durations of the light and dark period on path *i*, respectively. If *r*_1_ < *r*_2_, the organism should select path 1 provided it assesses the time-variant risk factors correctly. Of course, the time-scale of the light-dark cycles must be significantly faster than the time scale of adaptation, as the organism could otherwise simply adapt to every period separately and would not need to integrate the time-variant signal.

An interesting question is whether we potentially need to account for directional bias of the organism. Recently, *P. polycephalum* was found to exhibit chirality [23] in the search phase of the foraging, i.e. a directional preference when the plasmodium is *expanding*. In contrast to this, our suggested experiment investigates the *contraction* phase of foraging, i.e. the *shrinking* of the tube network. Previous studies [16] have not found a bias in the contraction phase in very similar set-ups. However, we cannot categorically exclude that it may occur in some experimental settings. We thus suggest to account for this possibility in the following way. In a preliminary phase our experiment is conducted without lighting to check for any directional bias. If, contrary to our assumptions, bias is found we can proceed in two ways. Firstly, we can modify the experiment to use a different geometry that eliminates directional differences: Three food sources are arranged such that a central source is located in the middle between two other sources. The plasmodium is restricted in the usual way to use two paths from the central source to the outer sources. The paths are shaped such that together they form a symmetric ‘S’-shape. Since both paths have the same left-right curvatures, we would expect this to eliminate bias. Any bias that cannot be eliminated can easily be accommodated in the numerical model by using a different thinning factor *δ*_*i*_ for each branch (Eq (5)) and fitting these to the experiment. We emphasise that it is unlikely that this is necessary, since previous studies have not found bias in the contraction phase [16].

### 2.3 Revised stochastic Tero-Kobayashi model

**Fig 3:**
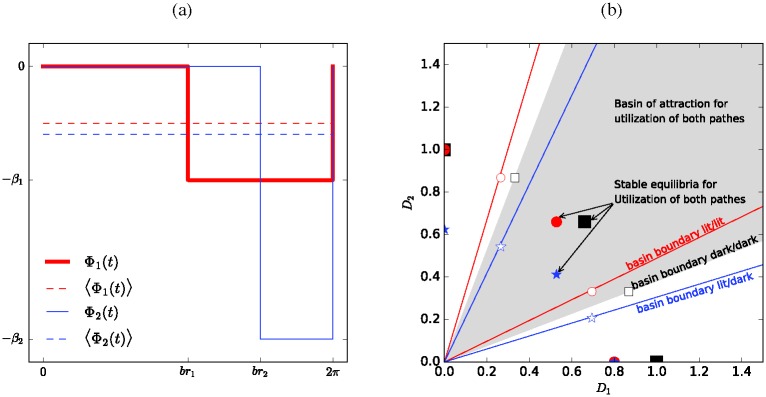
Influence of forcing. **a)** Forcing function Φ(*t*) for the modified deterministic Tero–Kobayashi model with intermittent lighting and parameters *br*_1_ < *br*_2_ and *β*_1_ < *β*_2_. Solid lines show the instantaneous forcing Φ_1_(*t*) (red) and Φ_2_(*t*) (blue), dashed lines are for the time-averaged forcing as denoted by the angle brackets 〈⋅〉. **b)** Attractors (markers) and basin of attraction (shaded) for the stable equilibrium with both *D*_1_ ≠ 0 and *D*_2_ ≠ 0 of the deterministic model. Squares are for the unforced model $\left(t\;\mathrm{mod}\;2\pi \right)<br_{1}$ (dark/dark), circles are for $br_{1}<\left(t\;\mathrm{mod}\;2\pi \right)<br_{2}$ (light/dark), stars are for $br_{2}<\left(t\;\mathrm{mod}\;2\pi \right)$ (light/light). Filled symbols denote stable equilibria, hollow symbols stand for unstable equilibria. The shaded region denotes the basin of attraction for the unforced model. The basins of attraction for the forced models are between the corresponding red lines (light/dark) and blue lines (light/light), respectively. The unshaded regions left/right respectively above/below these lines are the basins of attraction for convergence on a single path (*D*_1_ = 0 or *D*_2_ = 0). We assume here the model is locked forever into the respective forcing regime.

The proposed experiment corresponds to the following variation of the Tero-Kobayashi model restricted to two paths as given in Eq (6). As outlined above, a variation of lighting can be captured as a variation of *δ*. For intermittent lighting we introduce a forcing function

$$\begin{array}{c} \Phi \left(t,D_{i}\right)=\{\begin{array}{cc} -\beta_{i}D_{i} & \text{if}\;\left(\omega t\;\text{mod}\;2\pi \right)>br_{i}\\ 0 & \text{otherwise} \end{array} \end{array}$$ (7)

where *ω* is the frequency of the forcing, *br*_*i*_ is the length of the darkness period on path *i*, and *β*_*i*_ is a measure of the intensity of the lighting on path *i*. The lighting function is shown in Fig 3A.

The different behaviours of a system that remains forever in one of the three forcing regimes are shown in Fig 3B, which also gives the corresponding equilibria. The filled squares at (1/0), (0, 1), and ∼(0.7, 0.7) are the stable equilibria of a system in which both branches are continuously dark. Thus these are the states that such a system would attain in the long-run. Equivalently, circles mark the equilibria for an unchanging system in which only a single path is lit. Stars mark the equilibria for an unchanging system in which both paths are continuously lit. We direct the reader’s attention to how the equilibrium point that corresponds to equal use of both paths in the unlit regime moves away from the lit path(s). The corresponding basins of attraction (delineated with red lines for the light/dark regime and blue lines for the light/light regime) shift together with the stable equilibrium from their position in the completely unlit regime (grey shading). For reference, Fig 3 also shows with hollow markers the corresponding unstable equilibria. These are, however, not relevant to the long-run behaviour.

In each of the forcing regimes, the lighting can be taken into account as a modification of the tube thinning *δ*. If forcings are on a significantly faster time scale than the relaxation time of the system ($\frac{\omega }{2\pi }\gg \frac{1}{\delta }$), we may ignore the phase of the signal. Taking intensities into account, the time-averaged ratio of risks (ratio of forcings) is

$$\begin{array}{c} \rho =\frac{\beta_{1}}{\beta_{2}}\cdot \frac{2\pi -br_{1}}{2\pi -br_{2}}=\frac{\left\langle \Phi_{1}\left(t\right)\right\rangle }{\left\langle \Phi_{2}\left(t\right)\right\rangle } \end{array}$$ (8)

where the second path is preferred for *ρ* < 1 and the first one for *ρ* > 1.

As our aim is to analyze the role of noise in the assessment of time-variant risk, we introduce an explicit noise term *ξ*_*i*_(*t*) into the model. Here *ξ*_*i*_(*t*) is a Gaussian white noise process with an expected value of 〈*ξ*_*i*_(*t*)〉 = 0, unit variance 〈*ξ*_*i*_(*t*)*ξ*_*i*_(*t*)〉 ≡ *σ*^2^ = 1, and uncorrelated in time. This is also in line with the experimental finding that tube thicknesses fluctuate randomly to some degree.

The full model for the two path experiment with intermittent lighting becomes

$$\begin{array}{ccc} \frac{\partial D_{i}}{\partial t} & = & \left\{ \begin{array}{cc} F(D_{i}), & \text{if}\;D_{i}>0\\ 0, & \text{otherwise} \end{array}\right. \end{array}$$ (9)

$$\begin{array}{ccc} F(D_{i}) & = & -\delta D_{i}+f_{\epsilon }(\frac{D_{i}/l_{i}}{\sum_{j=1}^{2}D_{j}/l_{j}})+\Phi \left(t,D_{i}\right)+\sigma \xi_{i}\left(t\right) \end{array}$$ (10)

where we propose additive noise representing random perturbation originating from external sources.

We use the parameters in Table 1 with *l* ≡ *L*_1_ = *L*_2_ and *D*_1,0_ ≡ *D*_1_(*t* = 0) and similarly for *D*_2_. The risk ratio *ρ* is not a parameter, but is calculated from *br*_1_, *br*_2_, *β*_1_, *β*_2_. With *ω* ≫ *δ*, this establishes a process in which the forcing frequency is significantly faster than the relaxation time, but not so fast that—in the corresponding biological experiment—time averaging would happen on the sensory level of the organism. This combination of parameters implies a week preference of path 1 since *ρ* = 5/6 ≈ 1 and we expect that it is hard for the system to decide correctly.

**Table 1:** Experimental parameters.

General parameters								Forcing				
*L*_1_ = *L*_2_	*ϵ*	*δ*	*μ*	*σ*^2^	Δ*t*	$D_{1}^{0}$	$D_{2}^{0}$	*br*_1_	*br*_2_	*ω*	*β*_1_	*β*_2_
1	0.2	1	2	0.05	0.05	0.5	1	*π*	$\frac{3}{2}\pi$	10	0.25	0.6

## 3 Results

### 3.1 Integration of the modified Tero–Kobayashi model

Numerical integration of the modified model (Eqs (9) and (10)) with parameters as listed in Table 1 confirms that a well-attuned level of noise allows the system to decide correctly in respect to the time-variant risk. As a one-dimensional measure for the correctness of the decision we define

$$\begin{array}{c} c=\frac{D_{1}-D_{2}}{D_{1}+D_{2}}. \end{array}$$ (11)

For these parameters and assuming time-averaged risks, Path 1 is preferable to Path 2, because

$$\begin{array}{c} \rho =\frac{0.25}{0.6}\cdot \frac{2-1}{2-3/2}=\frac{5}{6}. \end{array}$$ (12)

Note that this risk ratio corresponds well to the ones found in biological experiments [19]. Thus, if the system decides “correctly” we expect lim_*t* → ∞_*c* → 1.

Disregarding forcing and noise, the system has three fixpoints (steady-states). Initialization determines which steady-state is attained. As we are interested in the question whether noise improves the decision behavior of the system, we initialize biased towards the wrong steady state with ($D_{1}^{0}=0.5,\;D_{2}^{0}=1,c=-1/3$). It turns out that the system decides correctly if a noisy process is assumed, while it will not be able to do so if noise is eliminated or reduced to a very low level.

**Fig 4:**
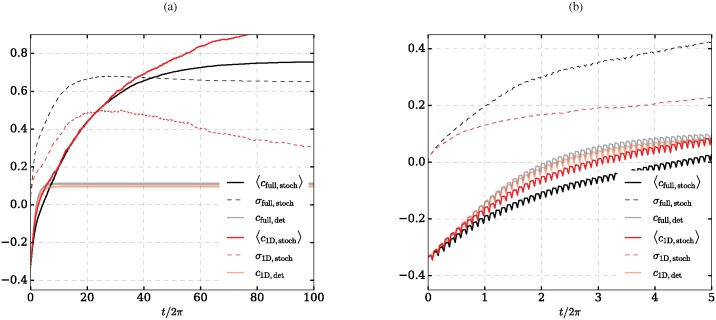
Comparison of system dynamics. Evolution of the mean correctness $c:=\frac{D_{1}-D_{2}}{D_{1}+D_{2}}$ and the variance *σ* for the full model (black/gray lines; subscript full) and the reduced one-dimensional one (red lines; subscript 1D). Opaque lines show the evolution of *c*_det_, i.e. for the corresponding deterministic system where *σ* = 0. (a) Poincaré section of the system for *ωt* = 2*πn* with n∈N, i.e. at the beginning of each forcing cycle; (b) complete time series for *t*/(2*π*) < 5, i.e. the first 50 forcing cycles.

The evolution of the noise-free deterministic process (Eqs (9) and (10)) for the parameters given above and *σ*^2^ = 0 is plotted in Fig 4A with a solid gray line. It is clearly visible that the process does not reach the correct decision *c* = 1, but instead assumes the third fixpoint (*D*_1_ ≈ *D*_2_ with *c* ≈ 0 but slightly shifted due to the forcing).

If noise is introduced into the system it does, however, decide correctly. To show this we compute 5000 sample paths of the stochastic process (Eqs (9) and (10)) with *σ*^2^ = 0.05 for over 2000 forcing cycles (200*π*) with 16 time steps per forcing cycle (Δ*t* = 2*π*/(16*ω*) ≈ 0.04) using the method of Milstein forward integration [24]. Fig 4B shows the mean and standard deviation for this process (refer to the bold continuous and bold dotted lines). It is clearly visible that the vast majority of samples decides correctly (*c* → 0.76). A further inspection of individual sample paths (not shown) reveals that none of them develops towards the state of no decision *D*_1_ ≈ *D*_2_ such that 88% of the realizations end up on the path imposing lower risk. This is due to the fact that noise destabilizes this fixpoint of the deterministic system.

### 3.2 Construction and analysis of a one-dimensional Itô Process

In principle, Markov theory offers us powerful means to further analyze this process. However, it is impossible (or at least extremely difficult) to apply such an analysis to the two-dimensional system (Eqs (9) and (10)) with a discontinuous forcing function. We thus aim to reduce it to a one-dimensional system that approximates the main properties of the full system well and is amenable to a formal analysis. A one-dimensional continuous-time continuous-space Markov process for the *c* value can be specified as an Itô-Diffusion

$$\begin{array}{c} \frac{dc}{dt}=\mu \left(c,t\right)+\sigma \left(c,t\right)\xi \left(t\right), \end{array}$$ (13)

where *μ* describes the deterministic development (so-called drift), and *ξ* is a Gaussian noise |*ξ*(*t*)| = 1, with mean 〈*ξ*(*t*)〉 = 0, and uncorrelated in time 〈*ξ*(*t*)*ξ*(*t*′)〉 = *δ*(*t* − *t*′). *σ* captures the fluctuation of the noise amplitude.

#### 3.2.1 Equation-free analysis

For a temporally homogeneous process we can attempt to infer a one-dimensional Itô-Diffusion from experimental data or simulation data by a technique known as equation-free analysis (EFA [25]). The idea is the following: We assume the existence of *μ*(⋅) and *σ*(⋅) and measure them from simulation data of the full system Eq (6). We then compare the evolution of *c*(*t*) measured from this simulation data with the evolution of *c*(*t*) obtained by forward integration of Eq (13) for a large number of sample paths. If these agree statistically, we are justified in our choice of *μ*(⋅) and *σ*(⋅) and can proceed by analysis of Eq (13) to understand the properties of the full system. We use a variant of EFA [15] that estimates drift and variance parameters for a process *X*(*t*) as:

$$\begin{array}{ccc} \mu (x) & = & \frac{\left\langle X(t+\delta t)-X(t)|X(t)=x\right\rangle }{\delta t} \end{array}$$ (14)

$$\begin{array}{ccc} \sigma^{2}\left(x\right) & = & \frac{\left\langle {\left[X\left(t+\delta t\right)-X\left(t\right)-\mu \left(x\right)\delta t\right]}^{2}|X\left(t\right)=x\right\rangle }{\delta t} \end{array}$$ (15)

where 〈⋅〉 denotes the sample average.

A complication is that the process under consideration is not temporally homogeneous, because of the forcing function, i.e. *μ* = *μ*(*c*, *τ*(*t*)) and *σ*^2^ = *σ*^2^(*c*, *τ*(*t*)) with *τ*(*t*) = *t* mod 2*π*. We thus divide the process into three different regimes corresponding to the three different forms of forcing: both paths dark; both paths lit; one path dark and one path lit. Note that the combination lit/dark only occurs in a single form with always the same path lit, depending on *br*_1_ and *br*_2_. Based on this we estimate the coefficients *μ*_*i*_(⋅), *σ*_*i*_(⋅) for each of the regimes *i* separately. Thus, assuming *br*_1_ < *br*_2_, each of the regimes by itself can be treated as a time-homogenous process.

$$\begin{array}{c} \frac{dc}{dt}=\{\begin{array}{cc} \mu_{1}\left(c\right)+\sigma_{1}\left(c\right)\xi \left(t\right), & \tau \leq br_{1}\\ \mu_{2}\left(c\right)+\sigma_{2}\left(c\right)\xi \left(t\right), & br_{1}<\tau \leq br_{2}\\ \mu_{3}\left(c\right)+\sigma_{3}\left(c\right)\xi \left(t\right), & \tau >br_{2} \end{array} \end{array}$$ (16)

**Fig 5:**
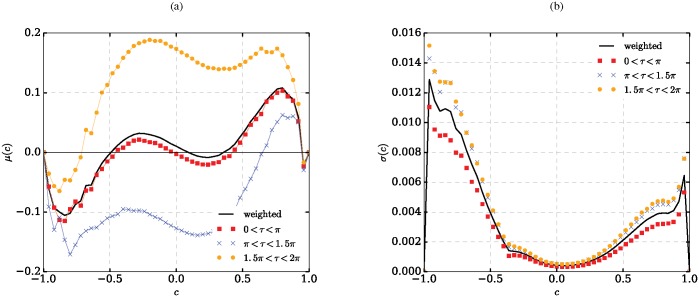
Equation-free analysis. EFA-estimated drift (a) and noise (b) coefficients of a one-dimensional temporally homogeneous Markov process for each of the three forcing regimes. In (b) the time-weighted average of all three forcing regimes is shown as well.

Fig 5 shows the results of EFA carried out with a bin size of 0.04.

In the first regime both tubes are unlit so that there is no forcing (0 ≤ *τ* < *π*). As one would expect based on the underlying deterministic process (Fig 2), the shape of the estimated drift function *μ*_1_(*c*) implies two locally stable equilibria, one globally stable equilibrium at the downgoing zero *c* ≈ 0, and two further globally stable equilibria at the absorbing interval boundaries (*c* ≈ ±1).

In the second regime only the first tube is lit ($\pi \leq \tau <\frac{3}{2}\pi$), which makes the second one more attractive. Thus the drift is shifted negative. The magnitude of the drift is increased due to the larger difference in risks between tubes.

In the third regime the first tube is more attractive ($\frac{3}{2}\pi \leq \tau <2\pi$; both tubes lit, but the second one more strongly than the first one). The drift is shifted to the positive region with the exception of the range *c* < −0.7 where the absorption of the boundary at *c* = −1 dominates. The dominating stable equilibrium is, however, the one at the interval boundary *c* = 1. Diffusion depends only weakly on the forcing regime.

#### 3.2.2 Simulation of the Itô process

We simulate the one-dimensional Markov process Eq (13) with *μ*, *σ* as obtained by EFA. Averages and variance for 500 sample paths are given in Fig 4B and compared to the full two-dimensional system. The figure shows that there is very good agreement between the expected values of the two processes. The variance follows the same pattern for both processes and is lower for the reduced system. The good agreement indicates that the essentially features of the process are captured in the reduced system and we may proceed with further analysis based on the reduced system.

#### 3.2.3 Governing Fokker–Planck equation and transition probabilities

**Fig 6:**
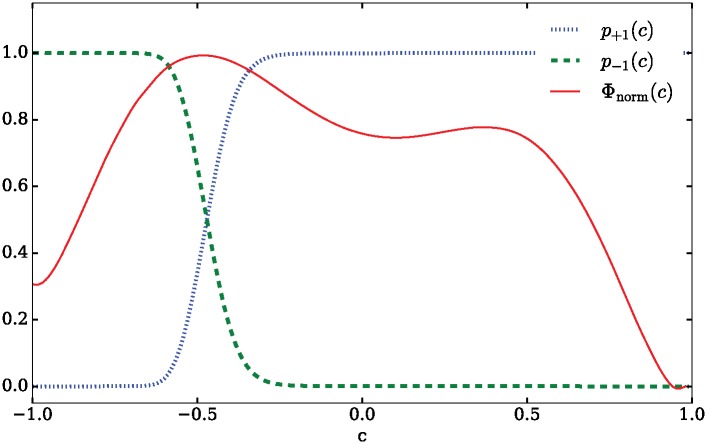
FPE potential. Φ_norm_ ≡ (Φ − min[Φ])/(max[Φ] − min[Φ]), and splitting probabilities *p*_±1_(*c*) of the temporally homogeneous process.

To this end, we construct a temporally-homogeneous Markov process by time-averaging the forcing regimes according to *br*_1_, *br*_2_. This is equivalent to a linear approximation of the influence of forcing *within each period*. A similar time-averaging could be achieved by performing an EFA assuming only a single (averaged) forcing regime for the whole process. Fig 6 gives the potential function Φ of this process [26]:

$$\begin{array}{c} \Phi \left(c\right):=-\int_{-\infty }^{x}\frac{2\hat{\mu }\left(s\right)}{{\hat{\sigma }}^{2}\left(s\right)}ds \end{array}$$ (17)

where $\hat{\mu }$ and ${\hat{\sigma }}^{2}$ are the time-averaged drift and variance. Local minima in Φ correspond to meta-stable points of the stochastic process and locally stable equilibria of the underlying deterministic process. It is clearly visible that the meta-stable point at *c* ≈ 0 has almost become a saddle due to the influence of noise. We are mainly interested in how likely it is that the system will evaluate the time-variant risk correctly and make a decision for Path 1.

This can be computed as the so-called splitting probability [24]. For a given interval [*a*, *b*], the splitting probability *p*_*a*_(*x*) gives the probability that the system initialized at *c* = *x* will reach the state *c* = *a* before the state *c* = *b*, i.e. in the case of a bi-stable process that it will make a decision for *a*. The corresponding probability *p*_*b*_(*x*) is defined symmetrically.

Let *f*(*t*, *y*) be the probability density for *c*(*t*) to take the value *y* at time *t*. The time-development of *f*(⋅, ⋅) is described by the Kolmogorov-forward or Fokker–Planck Equation (FPE [27]).

$$\begin{array}{c} \partial_{t}\;f\left(t,y\right)=-\partial_{y}\;[\hat{\mu }\left(y\right)\;f\left(t,y\right)]+\partial_{yy}\;[\frac{1}{2}{\hat{\sigma }}^{2}\left(y\right)\;f\left(t,y\right)] \end{array}$$ (18)

Its steady state *π*(*c*) = *f*(*c*, *t*) is time-independent, so that Eq (18) reduces to the ODE

$$\begin{array}{c} 0=-\frac{d}{dy}\;[\hat{\mu }\left(y\right)\;\pi \left(y\right)]+\frac{1}{2}\;\frac{d^{2}}{dy^{2}}\;[{\hat{\sigma }}^{2}\left(y\right)\;\pi \left(y\right)] \end{array}$$ (19)

We can thus calculate the steady state probability density function *π*(*x*) as the solution of Eq (19)

$$\begin{array}{c} \begin{array}{ccc} \psi (x) & = & e^{\int_{0}^{x}\left(2\hat{\mu }\left(y\right)/{\hat{\sigma }}^{2}\left(y\right)\right)dy}\\ \pi (x) & = & C\frac{\psi (x)}{{\hat{\sigma }}^{2}\left(x\right)} \end{array} \end{array}$$ (20)

where *C* is a suitable normalization constant [24]. Splitting probabilities for the process to leave the interval through the end at *c* = 1, i.e. with a correct decisions, can now be calculated for the PDF *π*(*x*) as [24, Eq (5.2.190)]

$$\begin{array}{c} p_{b}\left(x\right)=\int_{a}^{x}\psi \left(s\right)ds{\left(\int_{a}^{b}\psi \left(s\right)ds\right)}^{-1} \end{array}$$ (21)

The splitting probabilities are shown in Fig 6. The decision point lies at *c* ≈ −0.5 where *p*_+1_ = *p*_−1_. Thus, the system will decide correctly with *c* → 1 for most initialization points in the range. The transition from a wrong decision to a correct one is remarkably sharp considering that outside of the interval [−0.75, −0.25] the residual probability for the system to revise its decision is less than 0.001. From the potential Φ we know that the *c*-space projection of the attractor for the meta-stable point of no decision *D*_1_ ≈ *D*_2_ is −0.5 < *c* < 0.5. The expected probability of the process to decide correctly when initialized at a random position in this range is $\int_{-0.5}^{0.5}p_{+1}\left(c\right)dc=0.969$. In conclusion, the stochastic process will almost always decide correctly unless it is initialized with a very strong bias towards the wrong decision (*c* < −0.5). This is unlike the underlying deterministic process in which the decision depends almost entirely on the initialization and over a wide range of initializations no decision at all will be achieved.

Based on this analysis, we expect that the model can be tested with the proposed experiment in a straightforward fashion. We expect the success rate of the organism in the biological experiment to be significantly different from the one predicted by the *noise-free* model (*ξ* = 0), but we would expect these differences to disappear if *ξ* ≠ 0 is fitted to the data in a cross-validation procedure.

## 4 Discussion and conclusions

The mathematical analysis clearly shows that a well-attuned level of noise can enable the organism to correctly assess a time-variant risk, while the corresponding noise-free system fails to do so. This corroborates that noise plays a crucial functional role in self-organized systems. In biological terms, this is of evolutionary significance. Biological systems need to strike a delicate balance between flexible and stable behavior: Stability allows an organism or a group to concentrate its resources and to ignore irrelevant short-term fluctuations in the environment. Yet, adaptation in the case of stronger or more long lasting changes is required. A multi-stable behavior selection mechanism, such as the one analyzed here, can achieve exactly this by exploiting noise. It enables a self-organized system to reliably react to short-term changes in the environment while maintaining a generally stable behavior. The alternative of a control mechanism that follows every change in the environment would potentially be disadvantageous because it leads to unstable behavior.

Related findings have earlier been reported for ant colonies [8, 9]. There are two important differences between these studies and the present one. Firstly, the two biological systems investigated, ants and slime molds, are fundamentally different. Secondly, in the case of ants it was shown that noise enables them to react to changes in the environment by switching between multiple behaviors, whereas our study shows that noise can enable the slime mold to select the correct behavior in an environment that changes too frequently for it to follow individual changes. Instead of attempting to track each change, the organism can adopt a single behavior that maximizes the average long-term benefit. Yet, despite these differences both studies show that the decision making in ants and slime molds can be understood as instances of the same phenomenon: behavior selection as stochastic attractor switching [28].

In the case of ants, the effects have already been experimentally verified for the real biological system [9]. We have proposed a simple and concrete experiment to do this for *P. polycephalum*. Our mathematical analysis shows that this experiment will yield interesting outcomes whether it verifies our theoretical predictions or reveals that the model does not capture the full spectrum of decision making mechanisms in *P. polycephalum*.

Generally speaking, the fact that noise facilitates adaptive decision making is not tied to specific physical details of any particular biological system. Instead, it arises from very general mathematical properties of the underlying self-organized processes [10]. Mass recruiting ants and slime molds have very little in common biologically and physically. Yet, despite this, the phenomenological mathematical models that describe their behavior selection are very similar when constructed on the right level of observation. The same holds for a variety of other types of self-organized collective decision-making mechanisms in social organisms and human social systems. For example, food source selection [1] and clustering behavior [15] of honey bees, foraging patterns of bacteria [12], the emergence of fashion trends [13] and the dispersion of innovations [14] all can be and have been described with very similar mathematical models. This explains why some fundamental principles that govern self-organized collective behavior appear to be universal across the range [29, 30]. We may thus expect to also find similar beneficial effects of noise in other instances of self-organized decision making.

Noise has many origins. In any biological system two major influences are fluctuations in the environment and the stochastic nature of the underlying bio-chemical processes themselves. In the behavior of social groups, variations between individuals’ characteristics and the stochastic nature of interactions between group members provide additional sources of noise [9]. Pseudo-randomness in the form of deterministic chaos may also enter into the equation [15]. In fact, no real physical system is noise-free. Usually, however, we expect noise to be a disturbance and to degrade system performance or at best to be irrelevant. It is thus fascinating that evolution seems to have enabled some organisms to make constructive use of noise.

## Appendix A Analysis of the deterministic system

### A.1 Equilibria

Consider the system

$$\begin{array}{c} \frac{\partial D_{i}}{\partial t}=-\delta D_{i}+f(\frac{D_{i}}{D_{1}+D_{2}}) \end{array}$$ (22a)

with *f*(*Q*_*i*_) = (1 + *ϵ*)*Q*^2^/(*ϵ* + *Q*^2^) and *Q*_*i*_ = *D*_*i*_/(*D*_1_ + *D*_2_) for *i* = {1, 2}. In an equilibrium, it is ∂_*t*_*D*_1_ = ∂_*t*_*D*_2_ = 0 and hence

$$\begin{array}{c} \frac{1+\epsilon }{\delta }=\frac{\epsilon {\left(D_{1}+D_{2}\right)}^{2}+D_{1}^{2}}{D_{1}} \end{array}$$ (22b)

$$\begin{array}{c} \frac{1+\epsilon }{\delta }=\frac{\epsilon {\left(D_{1}+D_{2}\right)}^{2}+D_{2}^{2}}{D_{2}} \end{array}$$ (22c)

Substituting Eq (22b) into Eq (22c) delivers a sixth-order polynomial in *D*_2_, i.e. there are a maximum of six equilibria. The first three are easily determined

1) the trivial equlibrium with (*δD*_1_, *δD*_2_) = (0, 0)
2) one equilibrium on the *D*_2_ axis: (*δD*_1_, *δD*_2_) = (0, 1)
3) one equilibrium on the *D*_1_ axis: (*δD*_1_, *δD*_2_) = (1, 0)

Assuming that for the latter three *D*_1_ ≠ 0 and *D*_2_ ≠ 0, we may let *D*_2_ = *aD*_1_ where physical realizability implies $a\in R_{+}$. With *D*_2_ = *aD*_1_, Eqs (22b) and (22c) read as

$$\begin{array}{ccc} \delta D_{1} & = & f(\frac{D_{1}}{D_{1}\left(1+a\right)})=f(\frac{1}{1+a})=\frac{1+\epsilon }{\epsilon {\left(1+a\right)}^{2}+1} \end{array}$$ (23a)

$$\begin{array}{ccc} \delta aD_{1} & = & f(\frac{aD_{1}}{D_{1}\left(1+a\right)})=f(\frac{a}{1+a})=\frac{\left(1+\epsilon \right)a^{2}}{\epsilon {\left(1+a\right)}^{2}+a^{2}} \end{array}$$ (23b)

eliminating *D*_1_ from these equations delivers the following relation for *a* which is independent of *δ*:

$$\begin{array}{c} \frac{(1+\epsilon )a}{\epsilon {\left(1+a\right)}^{2}+1}=\frac{\left(1+\epsilon \right)a^{2}}{\epsilon {\left(1+a\right)}^{2}+a^{2}} \end{array}$$ (23c)

$$\begin{array}{c} \Rightarrow 0=\epsilon a^{3}+\left(\epsilon -1\right)a^{2}-\left(\epsilon -1\right)a-\epsilon \end{array}$$ (23d)

$$\begin{array}{c} \text{with}\;\text{the}\;\text{roots}\;a_{1}=1\;\text{and}\;\;a_{2/3}=\frac{1-2\epsilon }{2\epsilon }\pm \sqrt{\frac{2\epsilon -1}{2\epsilon }-1}. \end{array}$$ (23e)

The criterion for *a*_2/3_ to be real is $\epsilon <\frac{1}{4}$. Such that we find the three remaining equilibria

4) *a* = *a*_1_ = 1: $\left(\delta D_{1},\delta D_{2}\right)=(\frac{1+\epsilon }{1+4\epsilon },\frac{1+\epsilon }{1+4\epsilon })$
5) *a* = *a*_2_: $\left(\delta D_{1},\delta D_{2}\right)=(\frac{1+\epsilon }{\epsilon {\left(1+a_{2}\right)}^{2}+1},\frac{a_{2}\left(1+\epsilon \right)}{\epsilon {\left(1+a_{2}\right)}^{2}+1})$
6) *a* = *a*_3_: $\left(\delta D_{1},\delta D_{2}\right)=(\frac{1+\epsilon }{\epsilon {\left(1+a_{3}\right)}^{2}+1},\frac{a_{3}\left(1+\epsilon \right)}{\epsilon {\left(1+a_{3}\right)}^{2}+1})$

**Fig 7:**
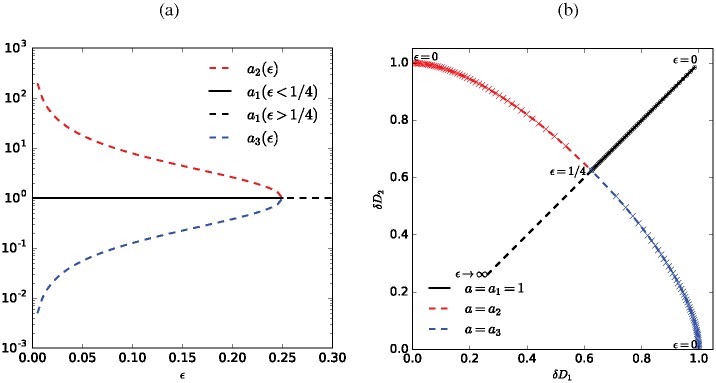
Linear stability. Solutions *a*_1_, *a*_2_ and *a*_3_ for the coefficient *a* linking *D*_1_ and the corresponding three equilibria for *ϵ* < 1/4. The symbols are equidistantly spaced in the range 0 < *ϵ* < 1/4 with Δ*ϵ* = 0.005. For *ϵ* ≥ 1/4, only one equilibrium exists which is shown by the dashed black line. Line style indicate the stability of the respective equilibrium where dashed corresponds to unstable and solid corresponds to stable equilibria.

These features are summarized in Fig 7.

### A.2 Linear stability of equilibria

The Jacobian of the System (22a) reads as

$$\begin{array}{c} J=(\begin{array}{cc} -\delta +\partial_{D_{1}}\left[f\left(Q_{1}\right)\right] & \partial_{D_{2}}\left[f\left(Q_{1}\right)\right]\\ \partial_{D_{1}}\left[f\left(Q_{2}\right)\right] & -\delta +\partial_{D_{2}}\left[f\left(Q_{2}\right)\right] \end{array}) \end{array}$$ (24a)

where we have (without summation over double-occurring indices)

$$\begin{array}{ccc} \partial_{D_{i}}\left[f\left(Q_{i}\right)\right] & = & 2\epsilon \left(1+\epsilon \right)\frac{D_{1}D_{2}\left(D_{1}+D_{2}\right)}{{\left[D_{i}^{2}+\epsilon {\left(D_{1}+D_{2}\right)}^{2}\right]}^{2}} \end{array}$$ (24b)

$$\begin{array}{ccc} \partial_{D_{1}}\left[f\left(Q_{2}\right)\right] & = & 2\epsilon \left(1+\epsilon \right)\frac{D_{2}^{2}\left(D_{1}+D_{2}\right)}{{\left[D_{2}^{2}+\epsilon {\left(D_{1}+D_{2}\right)}^{2}\right]}^{2}} \end{array}$$ (24c)

$$\begin{array}{ccc} \partial_{D_{2}}\left[f\left(Q_{1}\right)\right] & = & 2\epsilon \left(1+\epsilon \right)\frac{D_{1}^{2}\left(D_{1}+D_{2}\right)}{{\left[D_{1}^{2}+\epsilon {\left(D_{1}+D_{2}\right)}^{2}\right]}^{2}} \end{array}$$ (24d)

such that

$$\begin{array}{ccc} \frac{1}{\delta }J & = & \left(\begin{array}{cc} -1+2\frac{\epsilon }{\delta }\left(1+\epsilon \right)\frac{D_{1}D_{2}\left(D_{1}+D_{2}\right)}{{\left[D_{1}^{2}+\epsilon {\left(D_{1}+D_{2}\right)}^{2}\right]}^{2}} & 2\frac{\epsilon }{\delta }\left(1+\epsilon \right)\frac{D_{1}^{2}\left(D_{1}+D_{2}\right)}{{\left[D_{1}^{2}+\epsilon {\left(D_{1}+D_{2}\right)}^{2}\right]}^{2}}\\ 2\frac{\epsilon }{\delta }\left(1+\epsilon \right)\frac{D_{2}^{2}\left(D_{1}+D_{2}\right)}{{\left[D_{2}^{2}+\epsilon {\left(D_{1}+D_{2}\right)}^{2}\right]}^{2}} & -1+2\frac{\epsilon }{\delta }\left(1+\epsilon \right)\frac{D_{1}D_{2}\left(D_{1}+D_{2}\right)}{{\left[D_{2}^{2}+\epsilon {\left(D_{1}+D_{2}\right)}^{2}\right]}^{2}} \end{array}\right) \end{array}$$ (25)

#### A.2.1 Instability of the equilibrium (*δD*_1_, *δD*_2_) = (0, 0)

For small perturbations, the equilibrium is unstable along the axes. Consider a small perturbation *η* > 0 along the direction of *D*_1_ and no perturbation along *D*_2_ such that *Q*_1_ = 1:

$$\begin{array}{c} \frac{\partial D_{1}}{\partial t}=-\delta \eta +1>0 \end{array}$$ (26)

and we see that for small perturbations *η* < 1/*δ*, these perturbations continue to grow. The same holds for symmetry reasons along the *D*_2_ axis.

#### A.2.2 Stability of the equilibrium (*δD*_1_, *δD*_2_) = (1, 0)

The normalized Jacobian of the system is

$$\begin{array}{ccc} \frac{1}{\delta }J & = & \left(\begin{array}{cc} -1 & 2\frac{\epsilon }{\delta }\left(1+\epsilon \right)\frac{1}{D_{1}{\left[1+\epsilon \right]}^{2}}\\ 0 & -1 \end{array})=(\begin{array}{cc} -1 & \frac{\epsilon }{1+\epsilon }\\ 0 & -1 \end{array}\right) \end{array}$$ (27)

with eigenvalues −1 and −1. The system is hence stable with respect to small perturbations in on of the two variables. For symmetry reasons, the same must hold for the equilibrium (*δD*_1_, *δD*_2_) = (0, 1).

#### A.2.3 Stability of the equilibrium with *D*_1_ = *D*_2_

For $D_{1}=D_{2}=\frac{1+\epsilon }{1+4\epsilon }$, it is

$$\begin{array}{c} \partial_{D_{i}}\left[f\left(Q_{j}\right)\right]=2\epsilon \left(1+\epsilon \right)\frac{2D^{3}}{{\left[\left(1+4\epsilon \right)D^{2}\right]}^{2}}=\delta \frac{4\epsilon (1+\epsilon )}{{\left(1+4\epsilon \right)}^{2}}\frac{1+4\epsilon }{1+\epsilon }=\delta \frac{4\epsilon }{1+4\epsilon }\equiv \delta C \end{array}$$ (28)

$$\begin{array}{c} \frac{1}{\delta }J=(\begin{array}{cc} C-1 & C\\ C & C-1 \end{array}) \end{array}$$ (29)

with eigenvalues −1 and 2*C* − 1. The criterion for linear stability of the equilibrium is hence

$$\begin{array}{c} 2C-1<0\Rightarrow 8\epsilon <1+4\epsilon \Rightarrow \epsilon <0.25 \end{array}$$

### A.3 Summary of the stability features

In summary, we find that the bifurcation at $\epsilon =\frac{1}{4}$ is a sub-critical pitchfork bifurcation (cf. Fig 7):

- For *ϵ* < 1/4 (Fig 2a), the system has three basins of attraction. The margins of these basins of attraction in the *D*_1_–*D*_2_ phase space are given by the lines *D*_2_ = *a*_2_*D*_1_ and *D*_2_ = *a*_3_*D*_1_. These lines cannot be crossed by trajectories since for *a*_2_ and *a*_3_, it is also ∂_*t*_*D*_2_ = *a*∂_*t*_*D*_1_. The values of *a*_2,3_ depend on the parameter *ϵ* only (they are independent of *δ*). In between these two lines, the equilibrium *D*_1_ = *D*_2_ = (1 + *ϵ*)/(1 + 4*ϵ*) is attracted; trajectories originating elsewhere in the parameter space converge towards an equilibrium on one of the two axes.
- For *ϵ* > 1/4 (Fig 2b), the system has two basins of attraction. The margin between these two basins is given by the line *D*_1_ = *D*_2_ (which, is not crossed by any trajectory) and initial values on either side of this line converge to the respective equilibrium on that side. The equilibrium with utilization of both tubes continues to exist as a saddle point but looses its stability.

## Supporting information

S1 File Python script to reproduce the data used within this publication. In the supporting python script licensed under the GPL license, data used within this publication is generated or read from file.
(PY) Click here for additional data file.

S2 File Python script to reproduce figures used within this publication. In the supporting python script licensed under the GPL license, data used within this publication is generated or read from file and displayed in form of the figures shown within this publication.
(PY) Click here for additional data file.
